# To Lose Both Would Look Like Carelessness: Tasmanian Devil Facial Tumour Disease

**DOI:** 10.1371/journal.pbio.0040342

**Published:** 2006-10-17

**Authors:** Hamish McCallum, Menna Jones

## Abstract

How can you manage an emerging disease threat--in this case, Tasmanian devil facial tumor disease--that poses a serious conservation threat, when so little is known about the disease?

## Introduction

**Figure 1:**
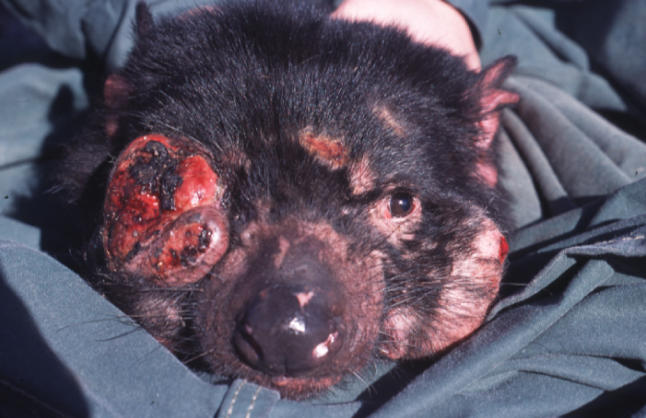
Tasmanian Devil Facial Tumour Disease (Photo: Menna Jones)

At the time of European settlement, Tasmania was the last remaining refuge of the two largest marsupial carnivores: the thylacine (or Tasmanian tiger), Thylacinus cynocephalus, and the Tasmanian devil, Sarcophilus harrisii. The extinction of the thylacine is perhaps the most notorious of the many Australian mammal extinctions since European colonisation. It has been partially blamed on disease [1], although there is little hard evidence to support this idea [2]. In 1996, Tasmanian devils were photographed in northeast Tasmania with what were apparently large tumours on their faces [3] (Figure 1). Sporadic reports continued during the next five years. By 2005, the tumours were occurring on more than half of the range of the species, and associated with substantial population declines. Following concerns that the disease might cause the extinction of the devil, the species has recently been listed as vulnerable to extinction at state and national levels. In the words Oscar Wilde put into Lady Bracknell's mouth, to lose one large marsupial carnivore may be regarded as a misfortune; to lose both would look like carelessness.

This paper uses the Tasmanian devil facial tumour disease (DFTD) as a case study of the wider issue of how to manage an emerging disease threat that poses a serious conservation threat: how should you proceed when you know very little? This is a question common to many ecological problems; all environmental management operates in the face of uncertainty [4]. If actions are postponed until higher-quality information is available, then it is likely that substantial costs will be incurred. Further, with emerging diseases or invasive species in general, it is likely that control will become more difficult or indeed impossible once the agent becomes established [5]. Rapid action is therefore essential but will inevitably be based on incomplete knowledge.

## What Is and Is Not Known?

DFTD appears to be a new disease that is restricted to devils. No affected animals were detected amongst the 2,000-plus devils trapped by six biologists between 1964 and 1995 [3]. Whilst neoplasms are quite common in dasyurids [6,7], there is no evidence of a similar cancer in any other Tasmanian mammal. Further, the tumour is sufficiently obvious (Figure 1) that it is inconceivable that it would not have been reported.

The apparent spatial and temporal progression of the disease [3] strongly suggests that it is infectious and that it is spreading. Transmission trials that are now under way should determine unequivocally whether it is infectious and provide an estimate of the incubation period. The identification of identical characteristic and complex chromosomal rearrangements in all tumours karyotyped, and the discovery of a devil that was heterozygous for a chromosome inversion that is homozygous in all tumours suggest that tumours may be transmitted directly between individuals as a rogue cell line (an allograft) [8]. Such a mode of transmission is known in one other infectious cancer: transmissible venereal sarcoma in dogs [9,10]. If this theory is correct, transmission probably occurs though biting. Transmission via tumour cells shed into carcasses or via vectors seems unlikely but cannot be unequivocally ruled out. The degree of infectivity of DFTD is poorly understood. Early indications are that it is not highly infectious. Despite individual devils being capable of moving up to 50 kilometres in one night, the disease appears to have taken three years to travel the 30 kilometres of the Freycinet Peninsula in eastern Tasmania [3]. In addition, DFTD does not appear to have spread into any captive populations, even in situations where there are adjacent affected wild individuals (H. Hesterman, personal communication), which suggests that transmission requires direct or very close contact. Confidence in this conclusion requires transmission trials and estimation of *R*_0_ (the number of secondary cases per primary case, when disease is rare). It is unclear whether resistance is developing and although the disease appears to have a genetic basis [8], the role of genetics and immunology in susceptibility or resistance is unknown.

Once the cancer becomes visible, it appears to be invariably fatal within a few months. The disease is rare in juveniles [3]. Nearly all devils appear to succumb between two (modal age of first breeding in females) and three years of age, resulting in very young age-structured populations in which most females are reduced to a single breeding event (from a mode of three) (M. Jones, A. Cockburn, C. Hawkins, H. Hesterman, S. Lachish et al., unpublished data). Populations where the disease has been present for several years appear to have declined by up to 80 percent, with as yet no evidence of either a cessation of decline or a diminution in the prevalence of disease [3] (S. Lachish, personal communication). There are signs of compensatory changes in the reproductive pattern of the animals following the appearance of the disease: there has been a three-fold increase in female devils breeding early, in their first year (M. Jones, A. Cockburn, C. Hawkins, H. Hesterman, S. Lachish et al., unpublished data)

 Anecdotal evidence is that devil numbers have been quite variable in the past century and that numbers about ten years ago were at historic highs [11]. Whilst a pattern of increases followed by collapses in the population size is consistent with the impact of density-dependent disease [12], it is also consistent with the action of a range of other density-dependent factors. Cessation of broad-scale strychnine poisoning for rabbits in the early 1950s [13] may also have led to a recent increase in population size. It is inconceivable that DFTD, which is so distinctive, had been responsible for previous reductions in population size.

## What Does Conventional Epidemiology Predict?

Disease has been responsible for the extinction of a number of species worldwide [14], but we know of no cases where a host-specific pathogen has driven its host entirely to extinction: there is usually at least one reservoir host upon which the pathogen has a limited effect and which can, therefore, provide a high force of infection onto the endangered species, even as the host declines towards extinction [2,15]. Given that any reservoir for DFTD appears unlikely, there is some cause to be optimistic about the likelihood of the disease itself not leading to extinction if transmission is density dependent. The pathogen should disappear once the host population drops below the threshold necessary for disease transmission, before host extinction [16]. However, empirical evidence for a wide range of pathogens suggests that transmission is rarely linearly dependent on density [17]. If the frequency of infected hosts in the population determines transmission rather than their density, there is no threshold population size. A pathogen may therefore be able to drive its single host species to extinction. The extent to which DFTD transmission might depend on host density is unknown. Biting is particularly associated with sexual behaviour in devils, and therefore the dynamics of the disease may resemble those of a sexually transmitted disease, in which case frequency-dependent transmission is to be expected [18]. Sources of mortality, which in the absence of the disease would not present a serious threat, may lead to extinction. For devils, these sources include road mortality [19], persecution, and habitat loss [20]. The prognosis for extinction risk may not be good.

## Possible Control Options

In principle, the elimination of an infectious disease from a population requires driving the basic reproductive rate *R*_0_ below one [21]. *R*_0_ can be reduced either by decreasing the rate of disease transmission per unit time or by reducing the time during which infected individuals are able to transmit infection.

Options therefore include: (1) reduction of rates of contact between infected and susceptible individuals, including quarantine and movement controls; (2) culling infected individuals; (3) culling all individuals in a given area; (4) vaccination or similar prophylactic treatment of uninfected individuals; (5) treating infected individuals; and (6) decontamination of the environment.

## An Agenda for Action and Research

**Figure 2:**
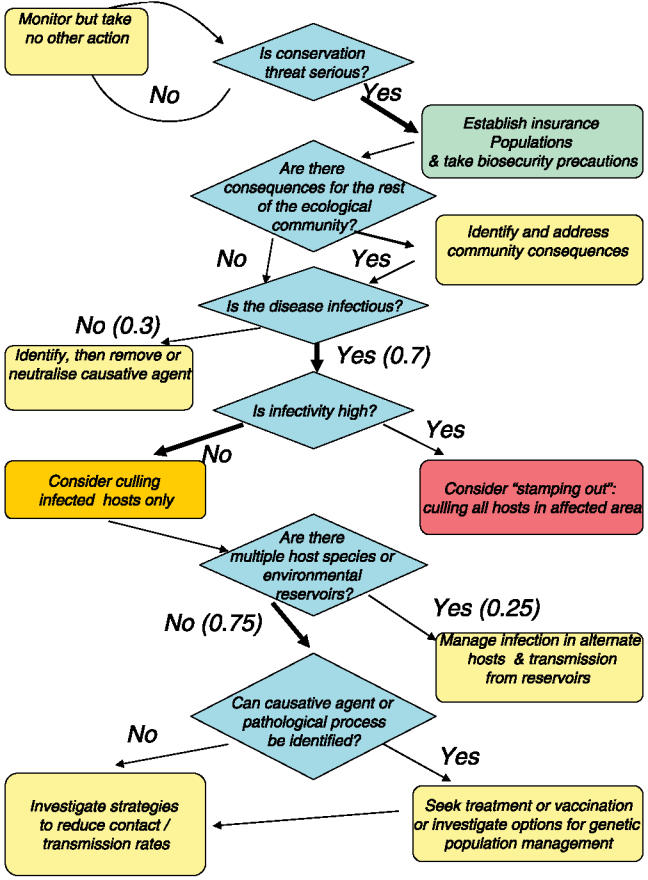
A Decision Tree for the Management of Emerging Wildlife Disease, with Particular Reference to Tasmanian Devil Facial Tumour Disease The relative thickness of arrows indicates the current likelihood of the given path representing the true situation. Probabilities determined by consensus of expert opinion at a recent technical workshop on DFTD [40] are shown in italics on the arrows. Colours represent the cost associated with the specified action, if it proves to be as a result of an incorrect decision. Red, high; yellow/orange, medium; green, low.

Figure 2 presents a decision tree for managing an emerging disease in wildlife. Given the uncertainties associated with an emerging disease, it is better to aim for a robust decision-making pathway that aims to maximize the chance of an acceptable outcome whilst maintaining flexibility to modify actions as more data become available [22,23], rather than seeking an optimal decision.

The first step is to determine whether the threat is severe enough to warrant action: “no action” is a valid management decision, but should be associated with ongoing monitoring of the situation. The obvious next step, especially if the conservation threat appears severe, is to attempt to establish disease-free captive and/or free-living populations in places that can be isolated from the disease. This approach may fail if vectors are involved, if the pathogen is highly infectious, or if the individuals transferred into such “insurance” populations are already infected but asymptomatic. For DFTD, the first two seem unlikely and the risk of the third can be managed.

The potential for the effects of disease to interact with the remainder of the ecological community must be assessed early; in some cases, this interaction may be more important than the direct effects of the disease on the focal species itself. Red foxes, Vulpes vulpes, have recently been introduced to Tasmania [24], and there is concern that reduced devil populations may permit foxes to become established, with the potential to cause the extinction of many mammals (including devils). The increased urgency of fox eradication does not rely on further knowledge about DFTD.

Whether or not the disease is infectious also requires an early decision, because it makes a fundamental difference to management, particularly whether removal of diseased animals is warranted. Recent examples of noninfectious diseases in the conservation literature include the decline in vultures on the Indian subcontinent attributable to residues of a veterinary drug [25], and widespread sea otter mortalities caused by domoic acid in algal blooms [26]. In these cases, the appropriate management action is to identify the factor (probably an environmental toxin) that induces disease and then to remove or neutralise it.

Crucially, the next decision point is to determine the degree of infectivity because of the extreme consequences of allowing a highly infectious disease to become established. If *R*_0_ is extremely high, which does not appear to be the case with DFTD, then the strategy of culling all individuals in the affected area (termed “stamping out” in the veterinary literature) may be an appropriate action. This is a standard approach used to control highly infectious diseases in livestock, such as foot-and-mouth disease [27]. For livestock, re-establishing the population may be expensive, but it is biologically straightforward. However, stamping out is a high-risk strategy for wild species. It will certainly increase the probability of extinction, at least on a local scale, and re-establishment is often difficult [28], with substantial issues relating to loss of genetic diversity. Further, attempting to eliminate the species over a substantial part of its current range would almost certainly be politically and ethically unacceptable as well as logistically extremely difficult. Whether broad-scale culling at an intensity less than total elimination of the local population would be successful is unknown without detailed knowledge of transmission dynamics. Such culling has been shown to be counterproductive in some cases, because it can lead to disruption of social organisation with increased movement and consequent increase in disease transmission [29].

For a moderately infective pathogen, culling only infected hosts (particularly in relatively closed populations) is likely to be a more acceptable and feasible management option. It may be less effective if there is a lengthy incubation period, because most infection may occur before the disease becomes apparent. However, if DFTD is indeed caused by interindividual transfer of tumour cells, it is unlikely that transmission will occur until the tumour grows to a size that is visible. The potential negative consequences of this strategy are much less than those of unselective culling; but, if infected individuals have some reproductive value, this value will need to be weighed against the benefit of removing them as potential sources of infection. “Learning by doing”, or adaptive management [30], with adequate replication and control sites is likely to be the only appropriate management strategy for implementing selective culling. Epidemiological models are central to evaluation of strategies throughout the decision process. However, delaying management decisions until sufficient data are collected to parameterise detailed population viability analysis–type models [31] is unwise.

The next decision point requires determining whether there are multiple hosts or a single host involved, whether there are vectors, and whether there are environmental reservoirs. Because there is a high level of confidence that DFTD is a single-host infection (Figure 2), we do not follow this branch in detail. In other, multiple-host systems, it is critical to manage infection in the reservoirs and transmission from the reservoirs to the species of conservation concern [32]. For example, the chytrid fungus Batrachochytrium dendrobatidis is associated with declines and extinctions in a wide variety of amphibian communities, and understanding the relative susceptibility to infection of different species and populations is essential [33].

In this decision tree, we have placed identification of the aetiological agent at a relatively late stage. Obviously, it is desirable to identify the causative agent of infectious disease, because it may open up a range of prophylactic or treatment options. There may be the possibility of treating infected individuals in captive situations. The canine transmissible sarcoma appears to be quite sensitive to standard cytotoxic drugs [34]. However, because these drugs require multiple intravenous treatments, they are not likely to be feasible for treating animals in the wild. If disease susceptibility (or resistance) is shown to be associated with particular genotypes, genetic management (artificial selection) could be incorporated into all aspects of management. Identification of the agent is neither sufficient nor necessary, however, for adequately managing a disease threat. For example, despite the frog chytrid fungus being identified as the causative agent of widespread amphibian mortality [35] almost ten years ago, we are little closer to managing (as distinct from studying) its impact on amphibian communities. None of the previous steps in the decision tree, any of which might be helpful in managing disease, absolutely requires the identification of the causative agent.

Evaluating the remaining potential control strategies, which focus on reducing contact and/or transmission rates within free-living populations, relies on estimating *R*_0_ and understanding something of its dependence on population density, social organisation and behaviour, and other ecological factors. There are at least three ways in which *R*_0_ might be estimated from field data. These include analysis of time-series data on increase in infection after the introduction of the disease to a new area [36], age-prevalence analysis in areas within which the disease is well established [37], and analysis of the rate of spatial spread [38]. Each of these, however, relies on knowledge of the incubation period.

From a theoretical perspective, whether the infection dynamics are density or frequency dependent is critically important in determining whether an infectious disease is likely to drive the host to extinction [14]. However, this can be investigated only by using field experiments. The dynamics of infection in any laboratory or captive situation may be entirely different, and transmission dynamics can be strongly influenced by social structure [39]. Such field experiments have proved extremely difficult [17].

The question of the nature of the transmission dynamics enters only at the final point of this decision tree. This information might be important in determining the long-term prognosis for the species survival, but it is unlikely to have much short- to medium-term impact on devising appropriate management strategies. Selective culling is likely to be far more effective than any attempts to control contact rates through manipulating food supply and feeding interactions, and the likely key periods for disease transmission during the mating season are outside human control.

## Abbreviations

DFTD: devil facial tumour disease
